# Coordinating efforts to optimize next-generation kidney replacement therapies: the European Kidney Health Alliance Work Group ‘Breakthrough Innovation’

**DOI:** 10.1093/ckj/sfad016

**Published:** 2023-01-27

**Authors:** Fokko Wieringa, Henning Søndergaard, Alberto Ortiz

**Affiliations:** IMEC, Holst Centre, Eindhoven, The Netherlands; UMCU, Utrecht, The Netherlands; EKHA WG3, Brussels, Belgium; EKHA WG3, Brussels, Belgium; Danish Kidney Association, Copenhagen, Denmark; EKHA WG3, Brussels, Belgium; University Autonoma of Madrid, Madrid, Spain

**Keywords:** chronic kidney disease (CKD), Decade of the Kidney, innovation kidney failure, kidney replacement therapy (KRT)

## Abstract

Chronic kidney disease (CKD) is one of the fastest growing health problems, set to become the fifth global death cause by 2040. Factors contributing to this fast growth include increased survival from other diseases (cancer, cardiovascular disease, diabetes, etc.), population aging, lack of early CKD diagnosis tools, insufficient CKD awareness within healthcare systems, limited therapeutic armamentarium to prevent CKD progression and limitations of currently available kidney replacement therapies (KRTs). The European Kidney Health Alliance (EKHA) and the American Association of Kidney Patients joined forces within the Decade of the Kidney^TM^ framework to address these issues. We report on the rationale and vision of the EKHA Work Group ‘Breakthrough Innovation’ which aims to disrupt the existing innovation paradox on KRT. We discuss how the concepts of international technological roadmapping and coopetition may leverage breakthrough KRT technologies, and present a map of the kidney innovation playing field, driven by patient advocacy groups.

## INTRODUCTION

Chronic kidney disease (CKD) is one of the fastest growing causes of death, set to become the fifth global cause of death by 2040 and the second by 2100 in those countries where life expectancy is longer [1]. The nature of the factors contributing to this fast growth is diverse. On one hand, advances in care driven by intensive research has increased survival from other diseases (cancer, cardiovascular disease, diabetes, etc.). On the other hand, population aging is driving up the prevalence of age-associated disease, such as CKD. However, there are also factors that are amenable to be corrected through research and awareness campaigns. These include the lack of tools for early diagnosis of CKD, insufficient awareness of the condition within healthcare systems, a limited therapeutic armamentarium to prevent CKD progression and limitations of current kidney replacement therapies (KRTs). A widely dispersed—but unfortunately wrong—perception is that for those in whom kidneys fail, the problem is ‘solved’ by dialysis or kidney transplantation, causing severe ‘underfunding’ of appropriate research [2]. The shocking truth is, however, that 5-year survival on dialysis is worse than for most cancer types [3]. The European Kidney Health Alliance (EKHA) and the American Association of Kidney Patients (AAKP) have joined forces within the Decade of the Kidney^TM^ framework to address these issues [4].

## THE ‘BREAKTHROUGH INNOVATION’ WORKGROUP IN EKHA

In 2021, EKHA established their ‘Breakthrough Innovation’ workgroup (EKHA WG3) with the aim to disrupt the existing innovation paradox on KRTs. EKHA WG3 is firmly inspired by medical doctors Willem Kolff, Belding Scribner, Carl Kjellstrand and Eli Friedman, but also by technologist Wayne Quinton, scientist/writer Arthur C. Clarke, physicist Richard Feynman and chemist/physicist Gordon Moore. This article pictures a pathway to climb upon the shoulders of these giants from multiple disciplines to re-kindle disruptive innovation for nephrology far beyond the limits of the presently possible.

## A HISTORICAL MEETING OF GIANTS

**Figure 1:**
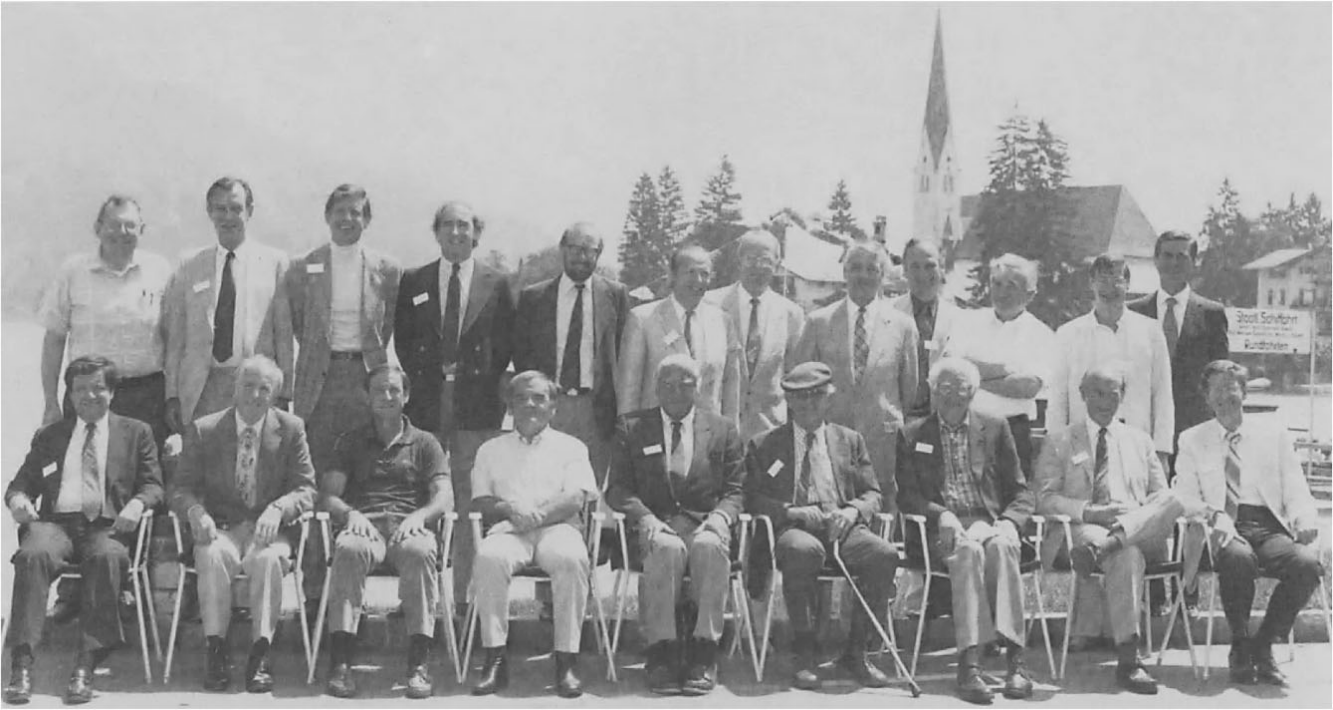
June 1986, Rottach-Egern, Germany: the world’s top nephrologists jointly formulate a future vision. From left to right, sitting: C.K. Colton, M.J. Lysaght, P.C. Farrell, S. Shaldon, G.E. Schreiner, B.H. Scribner, W.J. Kolff, R.H. Heptinstall, A.P. Lundin; standing: E.A. Friedman, J.F. Maher, L.W. Henderson, P.A. Keown, D.K. Peters, G. von Sengbusch, E. Quellhorst, H.J. Guriand, A.J. Wing, W. Schoeppe, C. Chantler, V. Bonomini [5].

In 1986, the small German village Rottach-Egern hosted a historical meeting (organized by the firm Enka) between all the ‘founding fathers’ of nephrology then still alive (Fig. 1).

All congress presentations and discussions, including a jointly formulated future vision from all these highly distinguished scientists, appeared in the 1988 published book *‘*Uremia Therapy—Perspectives for the Next Quarter Century’ [5]. This title would suggest that—by now—the book would only contain old news, but unfortunately this is not the case. We can very well still learn from it even now. The book contains an interesting list of what these experts then jointly saw as difficult but doable, titled ‘Ultimate goals of nephrology’:

- Arresting nephritis
- Pre-empting renal allograft rejection
- Utilization of xenografted kidneys
- Recycling nitrogen wastes in the gut
- Fabrication of a bionic kidney

Now let us keep this list in mind, while we introduce a scientist that became famous for his remarkably accurate technological visions for the future.

## THE THREE INNOVATION ‘LAWS’ OF CLARKE

Arthur C. Clarke (1917–2008) first worked as a British radar technician during WWII, then studied mathematics and physics at King’s College in London and quickly gained worldwide fame as a science fiction writer, while also being the inventor of geostationary communication satellites and an astonishingly accurate early predictor of several other technological developments that decades later indeed came true.

Clarke formulated three ‘laws’ that he applied for all his predictions on science and technology:

1st law: When a distinguished but elderly scientist states that something is possible, (s)he is almost certainly right. When (s)he states that something is impossible, (s)he is very probably wrong.
2nd law: The only way of discovering the limits of the possible is to venture a little way past them into the impossible.
3rd law: Any sufficiently advanced technology is indistinguishable from magic.

If we hold Clarke's 1st law against the above listed ultimate goals of nephrology, agreed upon as possible in 1986 by the worlds most distinguished nephrologists/inventors, then what went wrong? The Uremia Therapy book itself describes the answer: while initially the National Institutes of Health (NIH) successfully stimulated numerous artificial kidney milestones via the NIAMDD and NIH-AKCUP innovation programs, these programs began winding down in the 1970s and were completely terminated in 1980, ‘under the rationale that ESRD (end-stage renal disease) had by now achieved sufficient industrial stature to support its own research and development’ [5]. The book also states that ‘an industrial firm will make judgements on Research and Development (R&D) based on the expected return of the firm rather than on the overall value of the innovation for society’ and ‘private incentives may not generate the socially optimal amount or mix of R&D’. In other words: the funding needed to apply Clarke's 2nd law was progressively diminished and finally stopped. A likewise analysis was published in 2020 by Wieringa and Sheldon: ‘manufacturers may be reluctant to invest in innovative products. Moreover, why would they if present technologies are still good “cash-cows” that generate good profits, and growth markets are still emerging’, as well as ‘the present innovation paradox largely is a result of minimizing investment risks in a market where existing technologies are still very profitable’ [6]. Fortunately, they also pointed out two concepts that might help to break the innovation paradox: international technological roadmapping and coopetition. However, before diving deeper in these two terms, let us first look at two other famous scientists who also radically changed our world.

## FEYNMAN'S PREDICTION, APPLIED IN MOORE'S ‘LAW’

In 1960, physicist Richard Feynman published his famous article ‘There's plenty of room at the bottom’ in which he described the astounding potential of nanotechnology and the dazzling amount of information that could be stored if memory elements would approach the size of just a few atoms [7]. By 1965, chemist/physicist Gordon Moore applied Feynman's theoretical ideas to the emerging highly practical domain of integrated electronics—also known as integrated circuits (ICs or ‘chips’)—and predicted an exponential growth of the number of components per square millimetre of silicon chip surface, which became known as ‘Moore's law’, and ever since then has driven the electronic chip industry [8]. It became a self-fulfilling prophecy: although Moore's ‘law’ was just an extrapolation based upon the growth curve he observed until then, it provided enough conviction for investors to keep financing the breakthroughs needed to make the ‘law’ come true, up until the point where we presently indeed are nearing the atomic size limits, thus finally approaching ‘the bottom’ predicted by Feynman (while fortunately finding other solutions to keep pushing performance).

## INTERNATIONAL TECHNOLOGY ROADMAPPING

During this pursuit of Moore's law, the semiconductor industry was confronted with ever increasing costs to realize each next generation of production machines that could shrink the size of components further down, up to a point where a single firm could not finance the next step alone anymore. Thus, the field decided to compose an international technological roadmap, describing agreed-upon necessary milestones on the way forward, and joined forces on pushing the frontier within pre-competitive projects, while periodically updating their joint roadmap by building forth upon the realized progress. And this international semiconductor roadmap became an unprecedented success. Up until today, competing industrial parties and research institutes cooperate to achieve precompetitive milestones (nodes) for technologies that they all need to create their (mutually competing) products. The Kidney Health Initiative (KHI) recognized the potential of international technology roadmapping and adopted it to publish an innovation roadmap for KRTs [9]. Several EKHA members—the Dutch Kidney Foundation, European Renal Association (ERA), IMEC and others—contributed actively to this roadmap, and support its realization.

## COOPETITION

Jointly defining and especially jointly realizing an international technology roadmap, is a form of coopetition. Coopetition is described as: ‘a combination of collaboration and competition taking place in the context of a relationship, a business strategy, or a business model in which firms compete and cooperate with each other at the same time’ [10].

Coopetition goes beyond the old rules of competition versus cooperation to combine the advantages of both. Its rise is attributable to increasing interdependence between global players and the heightened need for collective action, risk sharing and strategic flexibility [11]. An increasing amount of research evidence makes it clear that coopetition can indeed be a successful driver of innovation [10].

Governments of different countries—and even whole geopolitical regions like the USA and European Union (EU)—can make bundling the best brains attractive via coopetition: they can use the KHI roadmap as a process for multiplicative knowledge sharing but keep its consensus process separate from the various funding mechanisms (which all are dilutive and vary by country or geopolitical region). Various funding parties—that all launch their own local calls for proposals within their own jurisdictions—can all point towards the same KHI roadmap and then encourage coopetition where there is synergy [6].

Various options to implement this might be considered: for example, one option might be giving proposal ranking systems the option to affix a fair number of extra points for truly synergetic proposals, even if they involve different jurisdictions (provided they match the jointly agreed roadmap).

International coopetition can help increase the chance that the best brains worldwide may efficiently work together. This approach already is being applied by the KidneyX innovation accelerator, a joint activity of the American Society for Nephrology (ASN) and the US government, which granted part of its prize money to the EU-US MI-TRAM consortium, which proposed to make evaluation kits of their MI-TRAM chip technology available to all parties with interest of possibly applying this technology within their own research trajectories [12]. The ‘try before you buy’ approach to provide evaluation kits for possible customers is very common within the chip industry, but it is new within the KRT innovation field. Yet, the willingness to share technology modules with other players (e.g. via non-exclusive licensing) can speed up innovation tremendously and simultaneously prevent ‘patent shelving’ [6]. But to make such a strategy optimally work, the various innovative parties must become mutually aware of their existence on the playing field.

## A MAP OF THE KIDNEY INNOVATION PLAYING FIELD

**Figure 2:**
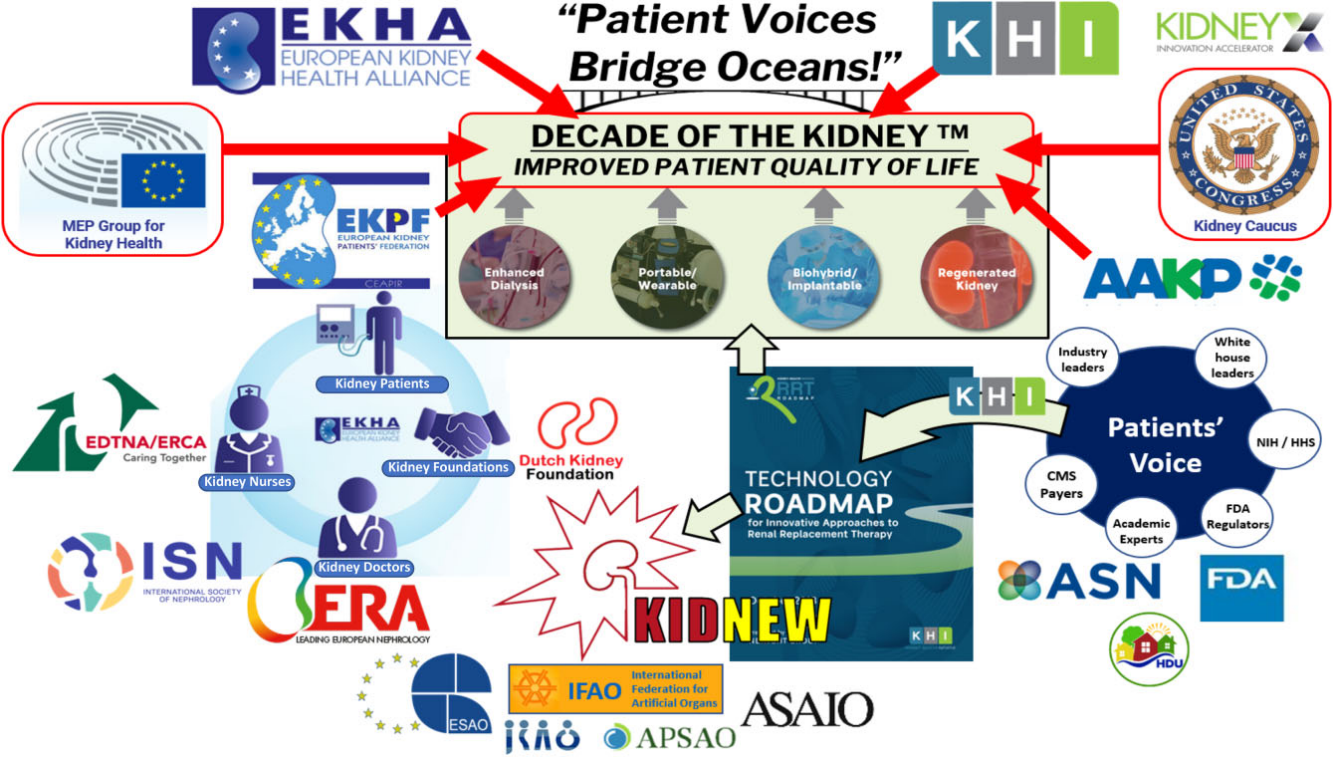
Organizations connected via the Decade of the Kidney™, the KHI technology roadmap and KIDNEW. With permission, reproduced (slightly updated) from reference [**13**]. Applied abbreviations: EKHA: European Kidney Health Alliance; KHI: Kidney Health Initiative; MEP: Member of European Parliament; EKPF: European Kidney Patient Federation; AAKP: American Association of Kidney Patients; EDTNA/ERCA: European Dialysis and Transplant Nurses Association—European Renal Care Association; NIH: National Institutes of Health; HHS: Human Health Service; CMS: Centers for Medicare and Medicaid Services; FDA: Food and Drug Administration; ISN: International Society of Nephrology; ERA: European Renal Association; ASN: American Society of Nephrology; HDU: Home Dialyzors United; ESAO: European Society of Artificial Organs; IFAO: International Federation of Artificial Organs; JSAO: Japanese Society for Artificial Organs; APSAO: Asian Pacific Society of Artificial Organs; ASAIO: American Society for Artificial Internal Organs; KIDNEW: Kidney Implant Developers Network.

At the 2021 congress Innovations in Dialysis: Expediting Advances Symposium (IDEAS) in Seattle, the EKHA ‘Breakthrough Innovation’ workgroup published a poster together with several other kidney-related innovators, that maps many organizations active in the field of kidney-related innovation (Fig. 2).

Figure 2 surely is not a complete map, but it does provide some interesting insights. It reveals several left/right similarities, ‘mirrored’ by the KHI roadmap: nephrologists, dialysis/transplantation nurses, patient consumers/advocacy organizations, and kidney foundations from Europe (united in EKHA) and the USA (united in KHI) are supported by their respective political representatives (EU Kidney Health MEP Group and US Congressional Kidney Caucus). The established worldwide structure of engineering societies for Artificial Organs (bottom row) is linked via KIDNEW. All are actively work on breakthrough R&D, supported by informed policy makers facilitating funding. The Technology Roadmap, initiated and maintained by KHI, plays a central role in coordinating the international efforts towards better and more affordable innovative kidney replacement therapies: KidneyX (top right) issues a prize competition for innovators providing plausible proposals to realize milestones described within the KHI innovation roadmap [4].

## PUTTING PATIENTS IN THE LEAD IS KEY

All the above information is nice but does not reveal how present innovation efforts might succeed where a solid plan by top experts failed in 1986. The crucial difference is putting patients in the lead and uniting their voice worldwide towards policy makers. This approach is taken by the AAKP, which started the Decade of the Kidney^TM^ movement and annually organizes a worldwide conference for kidney patients. The European Kidney Patients Federation (EKPF) and EKHA WG3 ‘Breakthrough Innovation’ jointly participate in this AAKP initiative, connecting members from all parties depicted in Fig. 2 and stirring ‘silos’ to boost multidisciplinary progress. We do this by facilitating direct discussions between experts and publishing the resulting insights [14], as well as by collating future predictions from experts in the field [15] all to contribute on fighting the unbearable lightness of neglecting kidney health [3] while reducing the eco-burden of KRTs [16]. During EKHA's 2022 European Kidney Forum at the European Parliament in Brussels, parliament members had a constructive open discussion with patients, nurses, doctors and technologists [17]. Likewise, the AAKP facilitates such discussions with the US Congressional Kidney Caucus on the American side.

EKHA WG3 stimulates practicing Clarke's 2nd law, while empirically translating findings to Clarke's 1st law. And, in the context of Clarke's 3rd law, we finally quote: ‘If you combine Physicians with Technicians, you get Magicians’ [13]. History shows this approach really works. On 4 April 1943 Dr Kolff connected Ms Janny Schrijver to his self-built machine and woke her up from uraemic coma [18]. In 1960 Scribner and Quinton realized the first arterio-venous shunt, enabling chronic haemodialysis. KRT-innovations then soared until governmental funding was shut down in 1980 [5]. After several decades, it is now time for a ‘Kolff and Scribner 2.0’ revival.
